# LINC01686 affects LPS‐induced cytokine expression via the miR‐18a‐5p/A20/STAT1 axis in THP‐1 cells

**DOI:** 10.1002/iid3.1234

**Published:** 2024-04-05

**Authors:** Jongwon Baek, Hyeung‐Seob Shin, Kyoungho Suk, Won‐Ha Lee

**Affiliations:** ^1^ School of Life Sciences, BK21 FOUR KNU Creative BioResearch Group Kyungpook National University Daegu South Korea; ^2^ Department of Pharmacology, Brain Science & Engineering Institute, BK21 FOUR KNU Biomedical Convergence Program Kyungpook National University School of Medicine Daegu South Korea

**Keywords:** inflammation, IκB‐ζ, LINC01686, macrophage, STAT1

## Abstract

**Background and Objective:**

Long noncoding RNAs (lncRNAs) are crucial in regulating various physiological and pathological processes, including immune responses. LINC01686 is a lncRNA with previously uncharacterized functions in immune regulation. This study aims to investigate the function of LINC01686 in lipopolysaccharide (LPS)‐induced inflammatory responses in the human monocytic leukemia cell line THP‐1 and its potential regulatory mechanisms involving miR‐18a‐5p and the anti‐inflammatory protein A20.

**Method:**

THP‐1 cells were stimulated with LPS to induce inflammatory responses, followed by analysis of LINC01686 expression levels. The role of LINC01686 in regulating the expression of interleukin (IL)‐6, IL‐8, A20, and signal transducer and activator of transcription 1 (STAT1) was examined using small interfering RNA‐mediated knockdown. Additionally, the involvement of miR‐18a‐5p in LINC01686‐mediated regulatory pathways was assessed by transfection with decoy RNAs mimicking the miR‐18a‐5p binding sites of LINC01686 or A20 messenger RNA.

**Results:**

LINC01686 expression was upregulated in THP‐1 cells following LPS stimulation. Suppression of LINC01686 enhanced LPS‐induced expression of IL‐6 and IL‐8, mediated through increased production of reactive oxygen species. Moreover, LINC01686 knockdown upregulated the expression and activation of IκB‐ζ, STAT1, and downregulated A20 expression. Transfection with decoy RNAs reversed the effects of LINC01686 suppression on A20, STAT1, IL‐6, and IL‐8 expression, highlighting the role of LINC01686 in sponging miR‐18a‐5p and regulating A20 expression.

**Conclusion:**

This study provides the first evidence that LINC01686 plays a critical role in modulating LPS‐induced inflammatory responses in THP‐1 cells by sponging miR‐18a‐5p, thereby regulating the expression and activation of A20 and STAT1. These findings shed light on the complex regulatory mechanisms involving lncRNAs in immune responses and offer potential therapeutic targets for inflammatory diseases.

## Graphical abstract

The role of long intergenic noncoding RNA 346 (LINC00346) in inflammation is understudied. Overexpression or knockdown of LINC00346 respectively suppressed or enhanced LPS‐induced NF‐κB activation and cytokine expression in THP‐1 cells. LINC00346 acts as an miR‐25‐3p sponge, promoting PTEN expression. PTEN inhibits PI3K‐mediated AKT/NF‐κB pathway activation. Notably, LINC00346 and PTEN were simultaneously downregulated in breast cancer tissues. Experiments in MDA‐MB‐231 cells confirmed the functional relationship between LINC00346, miR‐25‐3p, and PTEN during LPS‐induced NF‐κB activation. These findings highlight LINC00346's impact on PTEN/PI3K balance and downstream AKT/NF‐κB pathway in inflammation.

## 1 INTRODUCTION

Long noncoding RNAs (lncRNA) are transcripts longer than 200 bases and account for a significant fraction of non‐protein‐coding transcripts.^1^ LncRNAs are involved in physiological and pathological processes, including genomic imprinting, embryonic development, cell differentiation, tumor metastasis, and cell cycle regulation.^2^ Like messenger RNAs (mRNAs), lncRNAs are 5′‐capped, spliced, and polyadenylated. Evidence indicates that lncRNAs control cellular processes by interacting with RNA, DNA, and proteins.^3^, ^4^ Genome and transcriptome sequencing revealed about 21,000 protein‐coding genes against about 58,000 lncRNAs.^5^ The functions of lncRNAs are complex and diverse; they can regulate gene expression by competitively binding to common microRNAs (miRNAs), thus mitigating the inhibitory role of miRNAs on their targets.^5^ Although many lncRNAs have essential functions in various physiological and pathological processes, only a few have been reported to regulate the immune system.^2^ LINC01686 is a 2393‐nt‐lncRNA with an unknown function and is highly expressed in testes and ovaries.

A20, also known as tumor necrosis factor (TNF)‐α‐induced protein 3, is a potent anti‐inflammatory protein and a key regulator of apoptosis and the immune response. A20 is a ubiquitin‐editing enzyme with deubiquitinating activity and E3 ligase function.^6^ The function of A20 involves destabilizing important components of the nuclear factor kappa B (NF‐κB) signaling pathway by removing the polyubiquitin chains and promoting K48‐linked ubiquitination, leading to their degradation via the proteasome. Furthermore, A20 inhibits NF‐κB activity by interacting with receptor‐interacting serine/threonine kinase 1 (RIP1) and NF‐κB essential modulator (NEMO), ultimately leading to their degradation.^7^ Previous studies have demonstrated that A20 can inhibit the expression of signal transducer and activator of transcription 1 (STAT1), a major transcription factor involved in inflammation, and related genes in astrocytes and myeloid cells without affecting STAT3, independent of the NF‐κB pathway.^8^, ^9^, ^10^ Additionally, recent research has highlighted the role of miR‐18a‐5p, a known regulator of the pulmonary innate immune response, in downregulating the expression of A20.^11^, ^12^

The present study analyzed LINC01686 as a lncRNA implicated in macrophage activation. Results indicate that LINC01686participates in lipopolysaccharide (LPS)‐induced inflammatory activation of the human monocytic leukemia cell line THP‐1 by sponging a miR‐18a‐5p that sequentially regulates the expression and activation of A20 and STAT1.

## 2 MATERIALS AND METHODS

### 2.1 Antibody and reagents

The rabbit monoclonal antibodies (mAbs) against phospho‐NF‐κB p65 (Ser536), phospho‐STAT1 (Tyr701), phospho‐STAT3 (Tyr705), IκB‐ζ, and A20 and mouse mAb against STAT1 were purchased from Cell Signaling Technology. Mouse mAbs against p65 and β‐actin, rabbit mAb against phospho‐p65 (Ser276), and mito‐TEMPO were obtained from Santa Cruz Biotechnology. Bacterial LPS and N‐acetylcysteine (NAC) were purchased from Sigma‐Aldrich. DharmaFECT 1 small interfering RNA (siRNA) transfection reagent was obtained from Dhamacon. Scramble siRNA and LINC01686 siRNAs, A20 mRNA fragment, LINC01686 fragment, and miR‐18a‐5p inhibitor were provided by Bioneer. Tetramethylrhodamine, methyl ester (TMRM), MitoSOX Red, and Mitotracker Green were obtained from Invitrogen.

### 2.2 Cell culture

The human monocytic leukemia cell line, THP‐1, was grown in RPMI 1640 (WelGENE Inc.) supplemented with 10% fetal bovine serum, 0.05 mM β‐mercaptoethanol, glucose, and streptomycin‐penicillin at 37°C in 5% CO_2_.

### 2.3 Transfection of siRNA, decoy RNA, and miRNA inhibitor

THP‐1 cells (2.0 × 10^5^ cells) were pre‐seeded in six‐well plates with an antibiotic‐free culture medium. After 18 h, the culture medium was replaced with a fresh antibiotic‐free medium. Then, the cells were transfected with siRNA and decoy RNA (Table 1) and 100 nM of miRNA inhibitor using the DharmaFECT 1 siRNA transfection reagent according to the manufacturer's protocol. Transfected cells were collected and used for mRNA and protein analysis 48 h after transfection.

**Table 1:** Base sequences of the siRNA and decoy RNAs used in this study.

Name	Sequence
LINC01686 siRNA	5′‐GACUUAAAGAGAAAAAACUUUGU‐3′
A20 mRNA fragment	5′‐AUGCACCUUUC‐3′
LINC01686 fragment	5′‐CUGAUGCAAA‐3′

Abbreviations: mRNA, messenger RNA; siRNA, small interfering RNA.

### 2.4 Real‐time quantitative reverse transcription PCR (qRT‐PCR)

Total cellular RNA was extracted using TRIzol Reagent. Isolated RNAs were treated with RNase‐free DNase I (Takara Bio) and used for complementary DNA (cDNA) synthesis, which was conducted using the Reverse Transcription Master Premix (Elpis Biotech). qRT‐PCR was performed and analyzed using StepOnePlus (Applied Biosystems) with SYBR Premix Ex Taq (Takara Bio). The specific primer sequences are listed in Table 2. Glyceraldehyde 3‐phosphate dehydrogenase was used as a reference gene. Synthesis of cDNA from miR‐18a‐5p and quantification of miR‐18a‐5p transcript levels were performed using miRCURY LNA miRNA PCR Starter Kit (Qiagen) following the manufacturer's instructions. U6 was used as a control for normalization of miR‐18a‐5p.

**Table 2:** Base sequences of primers used in this study.

Gene	Primer	Sequence
LINC01686	F	5′‐TCCTACTCCACAACCAACGC‐3′
	R	5′‐TTGCCCAGTACAGCTTGGAG‐3′
TNF‐α	F	5′‐GGAGAAGGGTGACCGACTCA‐3′
	R	5′‐CTGCCCAGACTCGGCAA‐3′
IL‐6	F	5′‐GGTACATCCTCGACGGCATCT‐3′
	R	5′‐GTGCCTCTTTGCTGCTTTCAC‐3′
IL‐8	F	5′‐ATAAAGACATACTCCAAACCTTTC‐3′
	R	5′‐AAGCTTTACAATAATTTCTGTGTT‐3′
MCP‐1	F	5′‐ACTCTCGCCTCCAGCATGAA‐3′
	R	5′‐TTGATTGCATCTGGCTGAGC‐3′
STAT1	F	5′‐GCTCGTTTGTGGTGGAAAGAC‐3′
	R	5′‐TCACAGTGAACTGGACCCCT‐3′
STAT3	F	5′‐CTTTGAGACCGAGGTGTATCACC‐3′
	R	5′‐GGTCAGCATGTTGTACCACAGG‐3′
A20	F	5′‐CCACAAAGCCCTCATCGACAG‐3′
	R	5′‐GTCACCGTTCGTTTTCAGCG‐3′
Actin	F	5′‐TGAGATGCGTTGTTACAGGAAGTC‐3′
	R	5′‐GACTGGGCCATTCTCCTTAGAGA‐3′
GAPDH	F	5′‐TGGGCTACACTGAGC‐3′
	R	5′‐GGGTGTCGCTGTTGAAGTCA‐3′

Abbreviations: GAPDH, glyceraldehyde 3‐phosphate dehydrogenase; IL, interleukin; STAT1, signal transducer and activator of transcription 1; TNF, tumor necrosis factor.

### 2.5 Enzyme‐linked immunosorbent assay (ELISA)

Cytokine concentrations in culture supernatants were measured using ELISA Kits (Invitrogen). ELISA was performed according to the manufacturer's instructions. Colorimetric changes were detected using a microplate reader set at 450 nm (corrected by absorption at 540 nm). Measurements were performed in triplicate.

### 2.6 Western blot

The collected cell pellets were lysed using NP‐40 (IGEPAL CA‐630) lysis buffer (150 mM NaCl, 1% IGEPAL CA‐630, and 50 mM Tris [pH 8]) containing a protease inhibitor cocktail (Calbiochem) and a phosphatase inhibitor cocktail (Sigma‐Aldrich). The debris were removed from the whole cell lysate by centrifugation (12,000 rpm for 15 min at 4°C), and the remaining proteins were denatured with 100 mM DTT and heating. Protein samples were separated on a 10% polyacrylamide gel and blotted onto a polyvinylidene fluoride membrane (Milipore). Then, the membrane was incubated in a blocking solution (5% BSA in TBS containing 0.1% Tween 20 [TBS‐T]) for 1 h. The membrane was washed three times with TBS‐T and incubated with primary antibodies in blocking solution overnight at 4°C. Then, the membrane was washed three times with TBS‐T and incubated with horseradish peroxidase‐conjugated secondary antibodies at 4°C for 1 h. The membrane was washed with TBS‐T before chemiluminescence detection using a detection reagent (Corebio).

### 2.7 Flow cytometry

To measure the mitochondrial membrane potential or mitochondrial mass, THP‐1 cells (5.0 × 10^5^ cells) were suspended in Dulbecco's phosphate buffered saline (DPBS) and stained with 10 nM TMRM or 10 nM Mitotracker Green for 30 min at 37°C. For the detection of mitochondrial reactive oxygen species (ROS), cells were suspended in DPBS and treated with 5 μM of MitoSOX Red for 30 min at 37°C. After incubation, the cells were washed with DPBS, and the fluorescent intensity was analyzed by flow cytometry.

### 2.8 Statistical analysis

All data are presented as the mean values ± SEM. The statistical significance of differences between the means of the two groups was evaluated by one‐way analysis of variance or Student's *t*‐test using the GraphPad Prism Version 5.01. All experiments were conducted in triplicate, with the number of repetitions for each experiment detailed in the figure legends.

## 3 RESULTS

### 3.1 Inhibition of LINC01686 expression enhances LPS‐induced inflammatory gene expression

**Figure 1:**
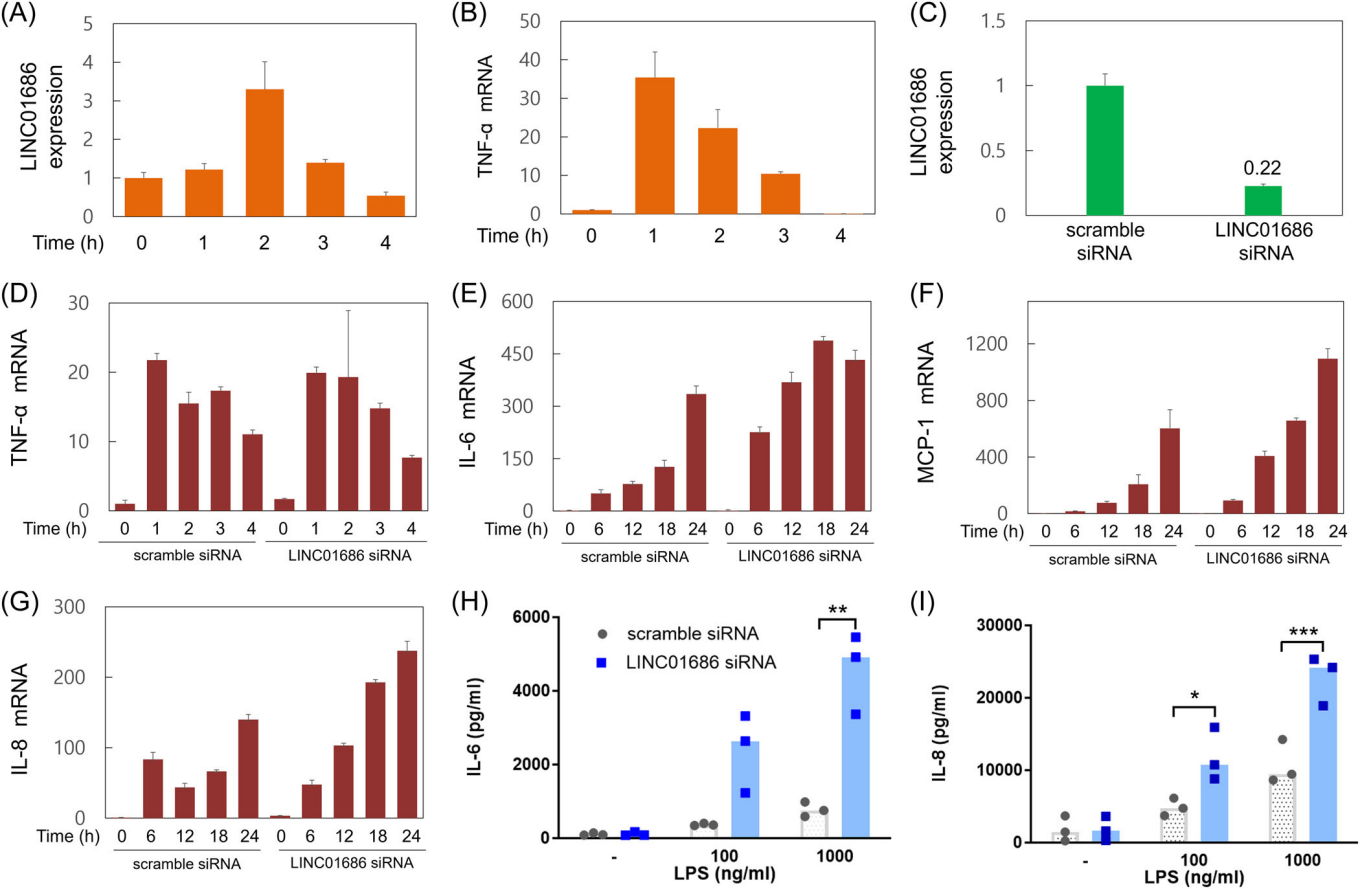
Attenuation of LINC01686 expression in THP‐1 cells enhances LPS‐induced cytokine expression. (A and B) THP‐1 cells were stimulated with LPS (300 ng/mL) for the indicated periods. The expression levels of LINC01686 and TNF‐ɑ mRNA were measured by qRT‐PCR. Numbers indicate the relative expression levels (*n* = 1). (C) THP‐1 cells were transfected with scramble siRNA or LINC01686 siRNA. After 24 h, the expression level of LINC01686 was measured by qRT‐PCR (*n* = 1). (D–G) THP‐1 cells were transfected with scramble siRNA or LINC01686 siRNA. After 48 h, the transfectants were stimulated with LPS (300 ng/mL) for the indicated periods. The levels of TNF‐ɑ, IL‐6, IL‐8, and MCP‐1 mRNA were measured by qRT‐PCR (*n* = 1). (H and I) THP‐1 cells were transfected with scramble siRNA or LINC01686 siRNA for 48 h. Transfectants were then stimulated with indicated doses of LPS for 24 h to measure IL‐6 and IL‐8 concentrations in the culture supernatant using ELISA (*n* = 3). *p < .05, **p < .01, ***p < .001. ELISA, enzyme‐linked immunosorbent assay; IL, interleukin; LPS, lipopolysaccharide; mRNA, messenger; qRT‐PCR, quantitative reverse transcription PCR; siRNA, small interfering RNA; STAT‐1, signal transducer and activator of transcription 1; TNF, Tumor necrosis factor.

To assess whether LINC01686 is expressed in the cells of the monocyte/macrophage lineage, THP‐1 cells were treated with LPS, and changes in LINC01686 expression levels were evaluated. The basal expression level of LINC01686 in THP‐1 cells increased upon LPS treatment (Figure 1A). The reactivity of THP‐1 cells to LPS was confirmed by quantifying TNF‐ɑ mRNA, whose expression level increased upon LPS treatment (Figure 1B).

Since the expression of LINC01686 increased under LPS stimulation in the macrophage‐like cell line THP‐1, LINC01686 might affect the LPS‐induced inflammatory response of macrophages. To identify the function of LINC01686 on LPS‐induced inflammatory responses in THP‐1 cells, we attenuated its expression in THP‐1 cells with a specific siRNA (siLINC01686) (Figure 1C), and the LPS‐induced expression of proinflammatory cytokine mRNAs was measured by qRT‐PCR. Attenuating LINC01686 expression did not affect the expression of TNF‐ɑ, whereas the expression of IL‐6, IL‐8, and MCP‐1 increased (Figure 1D–G). The enhancing effects of LINC01686 attenuation on LPS‐induced expression of IL‐6 and IL‐8 were also confirmed at the protein level by ELISA (Figure 1H,I).

### 3.2 Attenuation of LINC01686 expression induces mitochondrial dysfunction and ROS production

**Figure 2:**
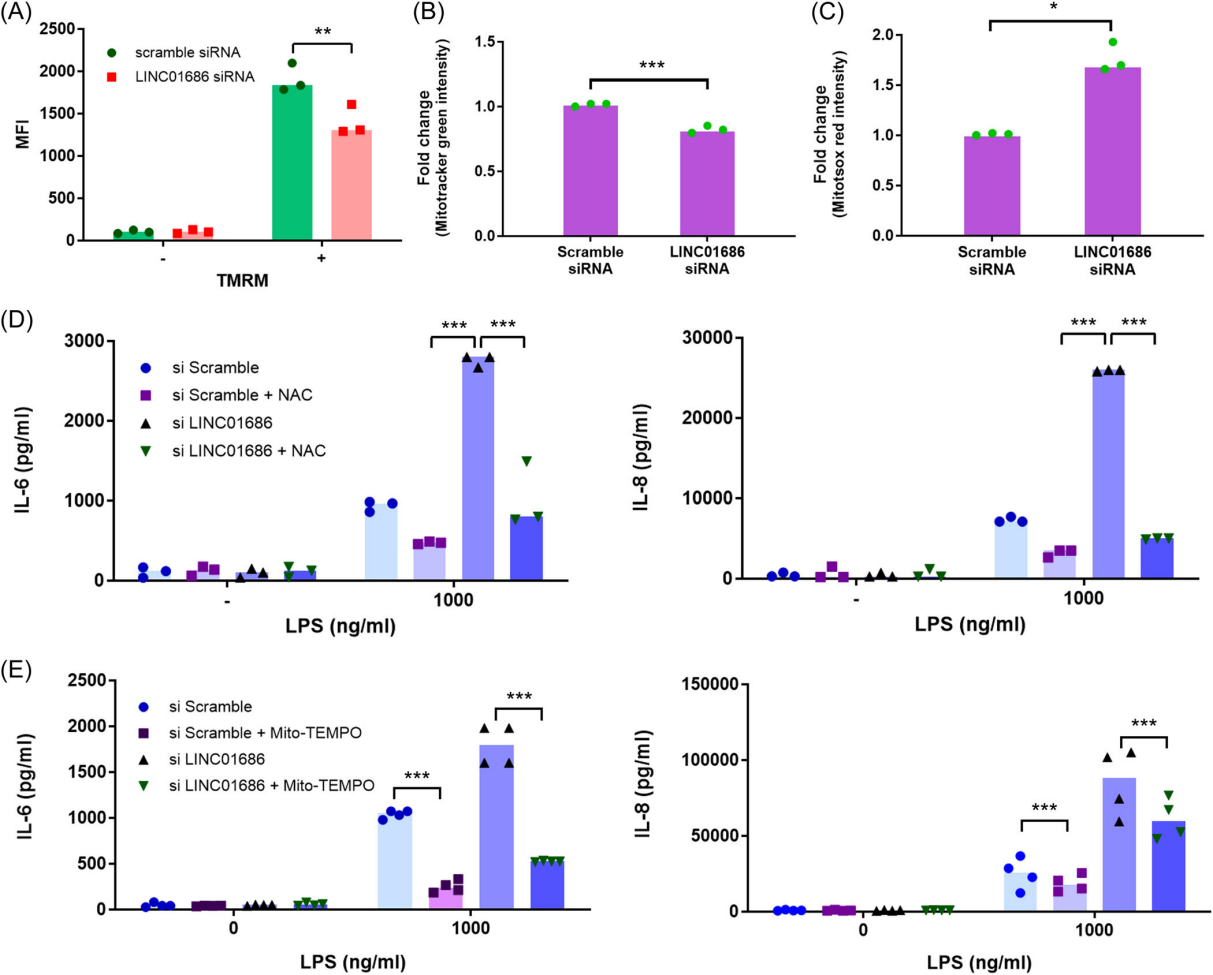
Attenuation of LINC01686 expression in THP‐1 cells disrupts the mitochondrial membrane potential and causes increased ROS production. (A–C) THP‐1 cells were transfected with scramble siRNA or LINC01686 siRNA for 48 h. Transfected THP‐1 cells were stained with TMRM (A) (*n* = 3), Mitotracker Green (B) (*n* = 3), or MitoSOX Red (C) (*n* = 3) for 30 min and analyzed by flow cytometry. (D) Cells were transfected as in A, and the transfectants were pretreated with NAC (15 mM) or PBS (3.75%) for 2 h before stimulation with LPS for 24 h. Concentrations of IL‐6 and IL‐8 in the culture supernatant were determined using ELISA (*n* = 3). (E) Cells were transfected as in (A), and the transfectants were pretreated with mito‐TEMPO (50 μM) or DMSO (0.1%) for 2 h before stimulation with LPS for 24 h. Concentrations of IL‐6 and IL‐8 in the culture supernatant were determined using ELISA (*n* = 4). *p < .05, **p < .01, ***p < .001. DMSO, Dimethyl sulfoxide; ELISA, enzyme‐linked immunosorbent assay; IL, interleukin; LPS, lipopolysaccharide; PBS, Phosphate buffered saline; siRNA, small interfering RNA; STAT‐1, signal transducer and activator of transcription 1.

Mitochondria are essential in cellular activities, and mitochondrial dysfunction promotes inflammatory activation, including the NF‐κB pathway.^13^ Therefore, to determine the effect of LINC01686 on mitochondrial function, mitochondrial membrane potential was measured using TMRM. Attenuating LINC01686 decreased the level of TMRM staining of intracellular mitochondria (Figure 2A). Since mitochondrial membrane potential affects ROS production, Mito Tracker Green—a total cellular mitochondrial mass detector—and MitoSOX Red—a mitochondrial ROS generation detector—were used to test the effect of LINC01686 attenuation on mitochondrial functions.^14^ Attenuating LINC01686 reduced the total mitochondrial mass per cell and increased mitochondrial ROS generation (Figure 2B,C). Treatment with NAC, an antioxidant, substantially reduced the enhancement of IL‐6 and IL‐8 secretion caused by LINC01686 attenuation but not completely in the case of IL‐6 (Figure 2D). Treatment with mito‐TEMPO, a mitochondria‐specific ROS inhibitor, had similar effects (Figure 2E). These results indicate that mitochondrial ROS production is partially involved in the enhanced cytokine secretion in cells upon LINC01686 attenuation.

### 3.3 LINC01686 attenuation enhances the expression and activation of STAT1 and IκB‐ζ

**Figure 3:**
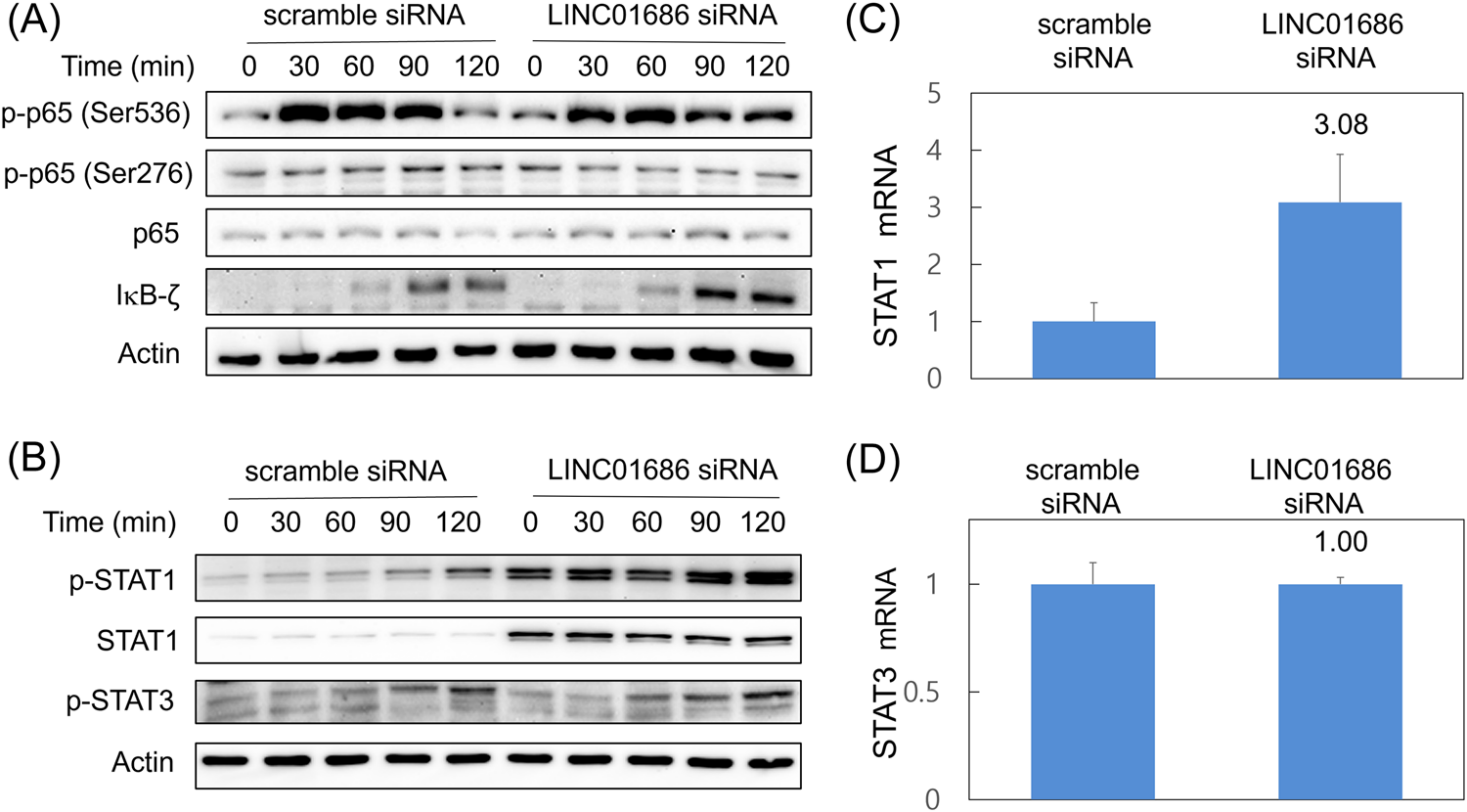
LINC01686 attenuation enhances expression and activation of STAT1 and IκB‐ζ. (A and B) THP‐1 cells were transfected with scramble siRNA or LINC01686 siRNA for 48 h. Transfectants were treated with LPS (1000 ng/mL) for the indicated periods. Western blot was performed using antibodies specific for phospho‐p65 (Ser^276^ or Ser^536^), p65, phospho‐IκB‐α, IκB‐α, β‐actin (A) (*n* = 1) or antibodies specific for phospho‐STAT1, STAT1, phospho‐STAT3, or actin (B) (*n* = 1). (C and D) Cells were transfected as in A and the expression levels of STAT1 and STAT3 were measured by qRT‐PCR. Numbers indicate the relative expression levels (*n* = 1). qRT‐PCR, quantitative reverse transcription PCR; siRNA, small interfering RNA; STAT‐1, signal transducer and activator of transcription 1.

To identify the molecular mechanisms responsible for the increased cytokine secretion caused by LINC01686 attenuation, the expression of transcription factors involved in IL‐6 and IL‐8 expression was investigated. NF‐κB is a major transcription factor regulating the expression of IL‐6 and IL‐8 in LPS‐activated macrophages. Phosphorylation of the NF‐κB p65 subunit is a critical factor required for its activation. In LPS‐stimulated THP‐1 cells, p65 expression and phosphorylation on Ser536 and Ser276 residues were not affected by LINC01686 attenuation (Figure 3A). NF‐κB activation may be regulated by factors other than p65 phosphorylation. IκB‐ζ selectively enhances or inhibits the expression of NF‐κB target genes and enhances the expression of *IL‐6*.^15^ Therefore, we examined the expression of IκB‐ζ to investigate whether the observed increase in IL‐6 expression was caused by IκB‐ζ. LPS‐induced expression of IκB‐ζ was increased by LINC01686 attenuation (Figure 3A).

STAT1 is involved in the regulation of IL‐6 and IL‐8 expression.^16^, ^17^ Additionally, activated STAT3 suppresses antitumor immunity^18^ and is known to act antagonistically to STAT1.^19^ In LPS‐stimulated THP‐1 cells, changes in the activation and expression of STAT1 and STAT3 after LINC01686 attenuation were examined. Results indicated that the activity of STAT3 was not affected by the attenuation of LINC01686, but the expression and phosphorylation of STAT1 were increased (Figure 3B). The expression level of STAT1 mRNA was also increased by LINC01686 attenuation, whereas that of STAT3 was not affected (Figure 3C,D). These results indicate that attenuation of LINC01686 enhances macrophage cytokine secretion through the upregulation and/or activation of IκB‐ζ and STAT1.

### 3.4 LINC01686 regulates STAT1 expression via the miR‐18a‐5p/A20 axis

**Figure 4:**
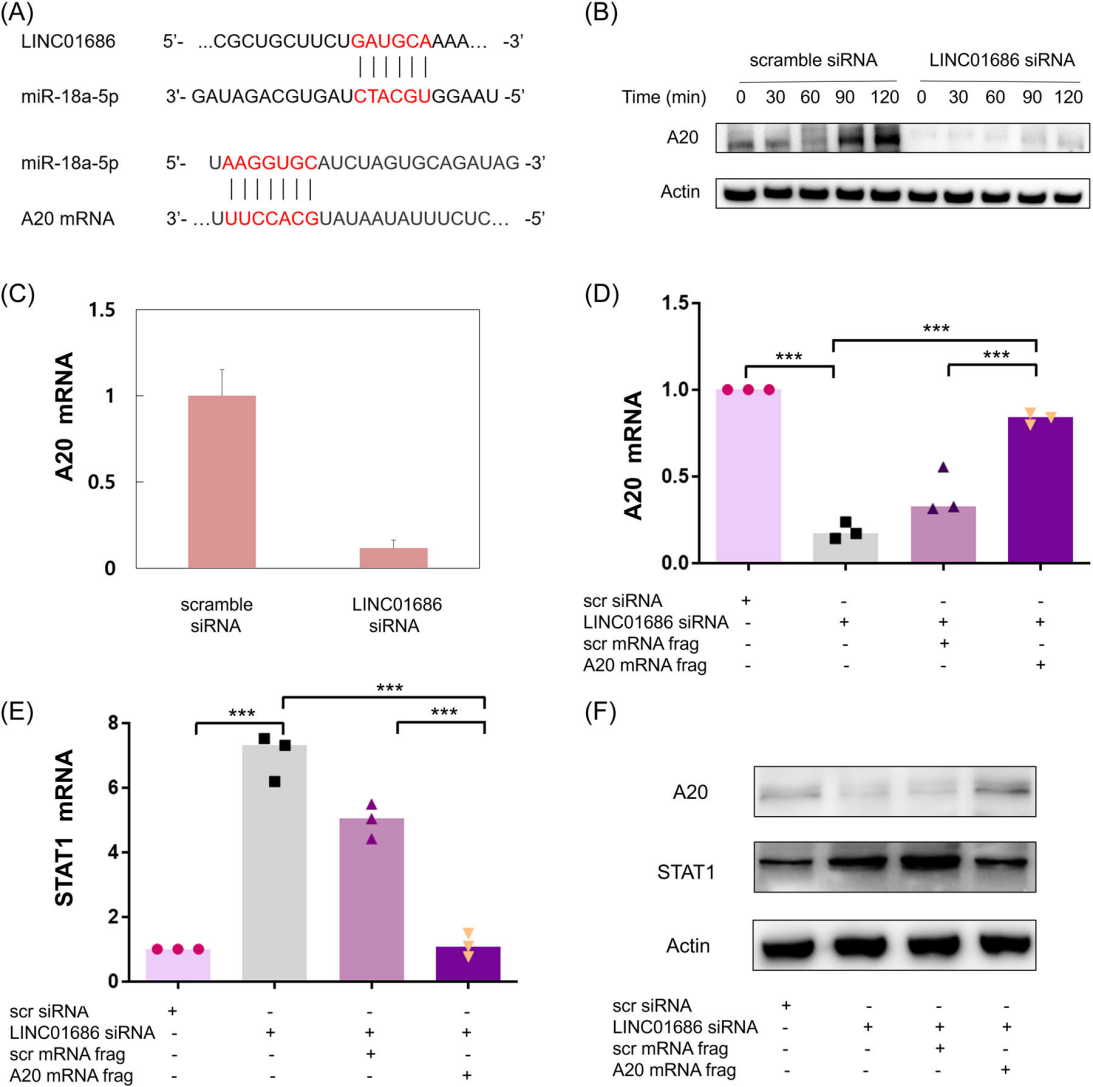
LINC01686 regulates STAT1 expression through the miR‐18a‐5p/A20 axis. (A) The putative miR‐18a‐5p binding sites in LINC0686 and A20 mRNA. (B and C) THP‐1 cells were transfected with scramble siRNA or LINC01686 siRNA for 48 h. Transfectants were treated with LPS (1000 ng/mL) for the indicated periods for quantification of A20 protein by western blot (B) (*n* = 1). For qRT‐PCR of A20 mRNA, cells were harvested 48 h after transfection (C) (*n* = 1). Number indicates the relative expression levels. (D–F) THP‐1 cells were transfected with LINC01686 siRNA or A20 mRNA fragments as indicated. After 48 h, expression levels of A20 mRNA (D) (*n* = 3) and STAT1 mRNA (E) (*n* = 3)were determined by qRT‐PCR, and the protein levels of A20, STAT1, and actin were evaluated by Western blot (F) (*n* = 1). ***p < .001. ELISA, enzyme‐linked immunosorbent assay; IL, interleukin; LPS, lipopolysaccharide; mRNA, messenger; qRT‐PCR, quantitative reverse transcription PCR; siRNA, small interfering RNA; STAT‐1, signal transducer and activator of transcription 1.

miRNAs are endogenous transcripts of less than 22 nucleotides and are critical factors regulating various cellular processes.^20^ The lead strand of miRNAs forms a complex with the RNA‐induced silencing complex, which complementarily binds to the target mRNA's 3′‐untranslated region and silences the transcript by inhibiting translation.^21^ One of the primary mechanisms by which lncRNAs regulate gene expression is by competitively binding to miRNAs to mitigate their gene‐repressive effects.^5^ To further illuminate the mechanism of STAT1 and IκB‐ζ activation by LINC01686 attenuation, the miRNAs targeted by LINC01686 and affecting the expression of STAT1 and IκB‐ζ were explored by searching databases, such as DIANA and miRDB. miR‐18a‐5p is one of the several miRNAs predicted to be the target of LINC01686 (Figure 4A). Although miR‐18a‐5p has oncogenic effects,^22^, ^23^, ^24^ it also inhibits A20,^12^ which inhibits STAT1 expression.^8^ Because LINC01686 attenuation was predicted to upregulate miR‐18a‐5p which then downregulates A20, we investigated the effect of LINC01686 attenuation on A20 expression. A20 expression level was increased in LPS‐stimulated THP‐1 cells, and LINC01686 attenuation suppressed A20 expression at the protein and mRNA levels (Figure 4B,C).

The decoy RNA technique was utilized to determine whether LINC01686 regulates A20 through miR‐18a‐5p. Decoy RNAs are artificial oligonucleotides with a binding site complementary to specific miRNAs and act as competitive inhibitors of target miRNAs.^25^ To investigate whether the inhibition of A20 expression was because of miR‐18a‐5p, a decoy RNA was designed using the putative miR‐18a‐5p binding site of the A20 mRNA (Figure 4A). The A20 decoy RNA mitigated the suppression of A20 and the enhancement of STAT1 caused by LINC01686 attenuation at the mRNA and protein levels (Figure 4D–F).

**Figure 5:**
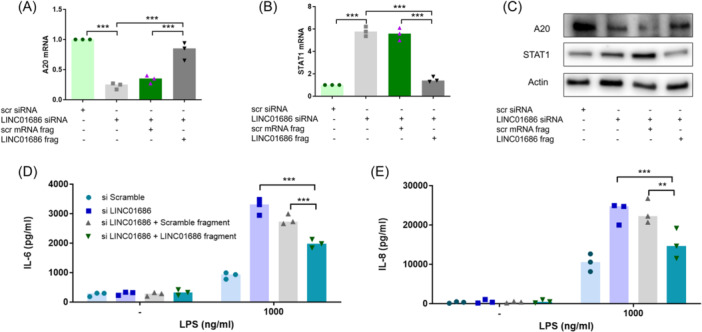
LINC01686 regulates LPS‐induced expression of IL‐6 and IL‐8 via the miR‐18a‐5p/A20/STAT1 axis. (A–C) THP‐1 cells were transfected with LINC01686 siRNA or LINC01686 fragments as indicated. After 48 h, expression levels of A20 mRNA (A) (*n* = 3) and STAT1 mRNA (B) (*n* = 3) were determined by qRT‐PCR, and the protein levels of A20, STAT1, and actin were evaluated by Western blot (C) (*n* = 1). (D and E) Cells transfected as in A were stimulated with LPS for 24 h, and the production levels of IL‐6 and IL‐8 in the culture supernatant were determined by ELISA (*n* = 3). **p < .01, ***p < .001. ELISA, enzyme‐linked immunosorbent assay; IL, interleukin; LPS, lipopolysaccharide; mRNA, messenger; qRT‐PCR, quantitative reverse transcription PCR; siRNA, small interfering RNA; STAT‐1, signal transducer and activator of transcription 1.

A LINC01686 decoy RNA, representing the miR‐18a‐5p binding site of LINC01686, also mitigated A20 inhibition and STAT1 enhancement caused by LINC01686 attenuation at the mRNA and protein levels (Figure 5A–C). The increased levels of IL‐6 and IL‐8 caused by LINC01686 attenuation was also suppressed by the LINC01686 decoy RNA (Figure 5D,E). The increase in IL‐8 levels was completely abolished, whereas that of IL‐6 showed partial relief, presumably due to the enhancement of IL‐6 secretion being affected by STAT1 and IκB‐ζ.

**Figure 6:**
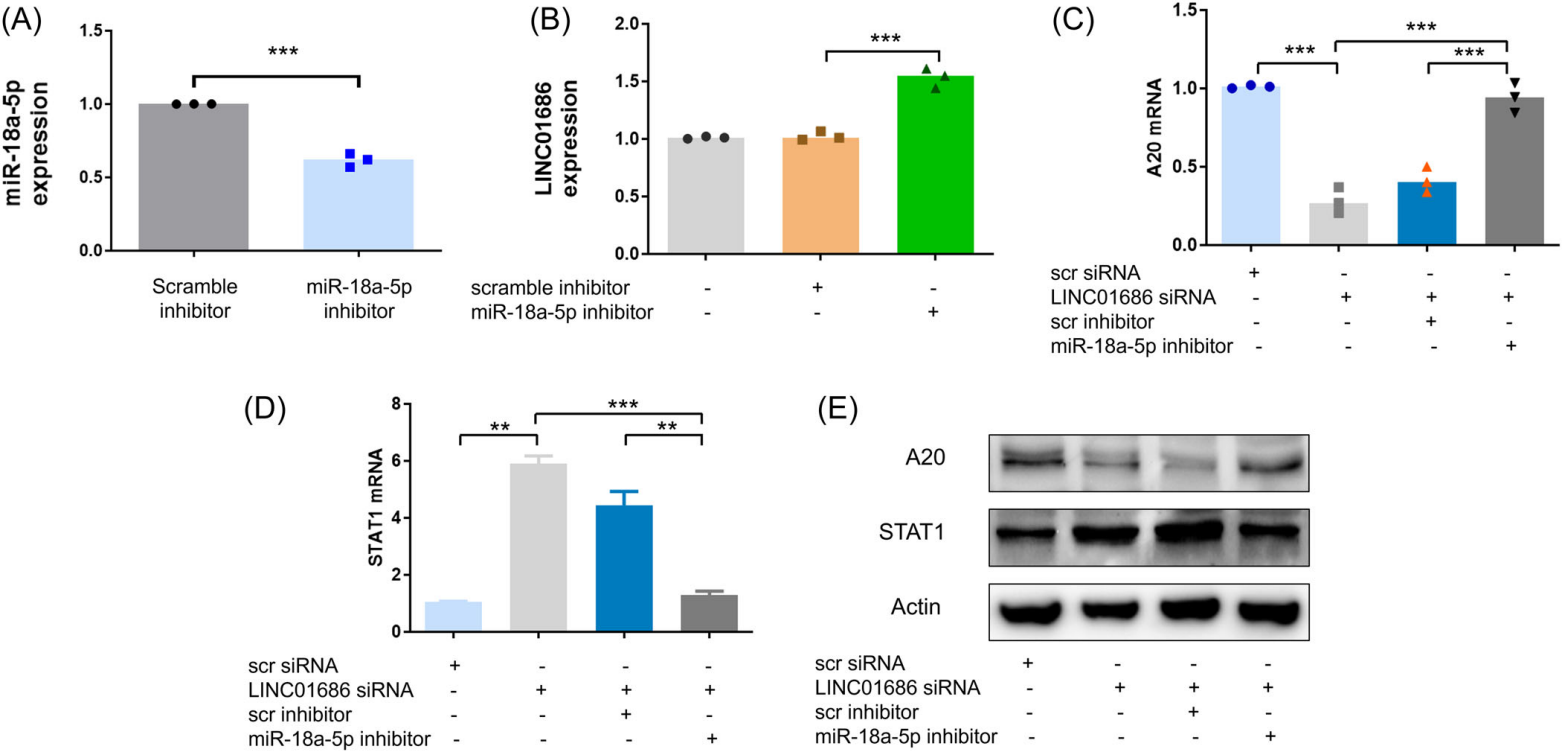
LINC01686 regulates the expression of A20 through miR‐18a‐5p. (A and B) THP‐1 cells were transfected with scramble or miR‐18a‐5p inhibitor as indicated. After 48 h, the expression level of miR‐18a‐5p (A), LINC01686 (B) was determined by qRT‐PCR (*n* = 3). (C–E) THP‐1 cells were transfected with LINC01686 siRNA or miR‐18a‐5p inhibitor as indicated. After 48 h, expression levels of A20 mRNA (C) and STAT1 mRNA (D) were determined by qRT‐PCR (*n* = 3), and the protein levels of A20, STAT1, and actin were evaluated by Western blot (E) (*n* = 1). **p < .01, ***p < .001. mRNA, messenger; siRNA, small interfering RNA; STAT‐1, signal transducer and activator of transcription 1.

To confirm the functionality of the LINC01686/miR‐18a‐5p/A20 axis, THP‐1 cells were treated with an miR‐18a‐5p inhibitor, and the expression levels of LINC01686 and A20 were tested. The inhibition of miR‐18a‐5p in THP‐1 cells increased the expression levels of LINC01686, confirming the interaction between LINC01686 and miR‐18a‐5p (Figure 6A,B). The miR‐18a‐5p inhibitor also mitigated the A20 inhibition and STAT1 upregulation caused by LINC01686 attenuation at the mRNA and protein levels (Figure 6B–D). These results indicate that the miR‐18a‐5p sponging function of LINC01686 regulates the expression of A20 and consequently STAT1.

## 4 DISCUSSION

The present data demonstrate for the first time the possible immunomodulatory activity of LINC01686 in monocyte/macrophage function. As attenuation of LINC01686 expression enhances LPS‐induced cytokine expression, the original function of LINC01686 appears to limit the magnitude of macrophage response to LPS by suppressing excess ROS and cytokine production. As LPS induces the expression of LINC01686, it indicates that this lncRNA is a negative feedback regulator of the inflammatory activation of monocytes and, possibly, macrophages.

As a component of the defense system against infection, tissue damage, and exposure to harmful chemicals, inflammatory cells identify the affected tissue, recruit more white blood cells into the area, remove the problematic substances, and repair damaged tissues.^26^, ^27^, ^28^ The inflammation process involves interactions between cells and inflammatory mediators, such as cytokines, chemokines, and cell surface proteins. Cytokines activate innate and acquired immunity, and the inflammatory response is a state where cytokines are continuously elevated from infection in external tissues or damage in internal tissues.^28^, ^29^ However, inappropriately activated or overactive inflammation can be harmful because these responses may contribute to the development of diseases, such as atherosclerosis, systemic lupus erythematosus, and rheumatoid arthritis.^27^, ^28^ In this respect, investigating the proper regulation of inflammatory cells, particularly macrophages, has been crucial. Our data may provide methods for macrophage activity regulation that could be used for the treatment of the pathogenic conditions associated with acute and chronic inflammation.

**Figure 7:**
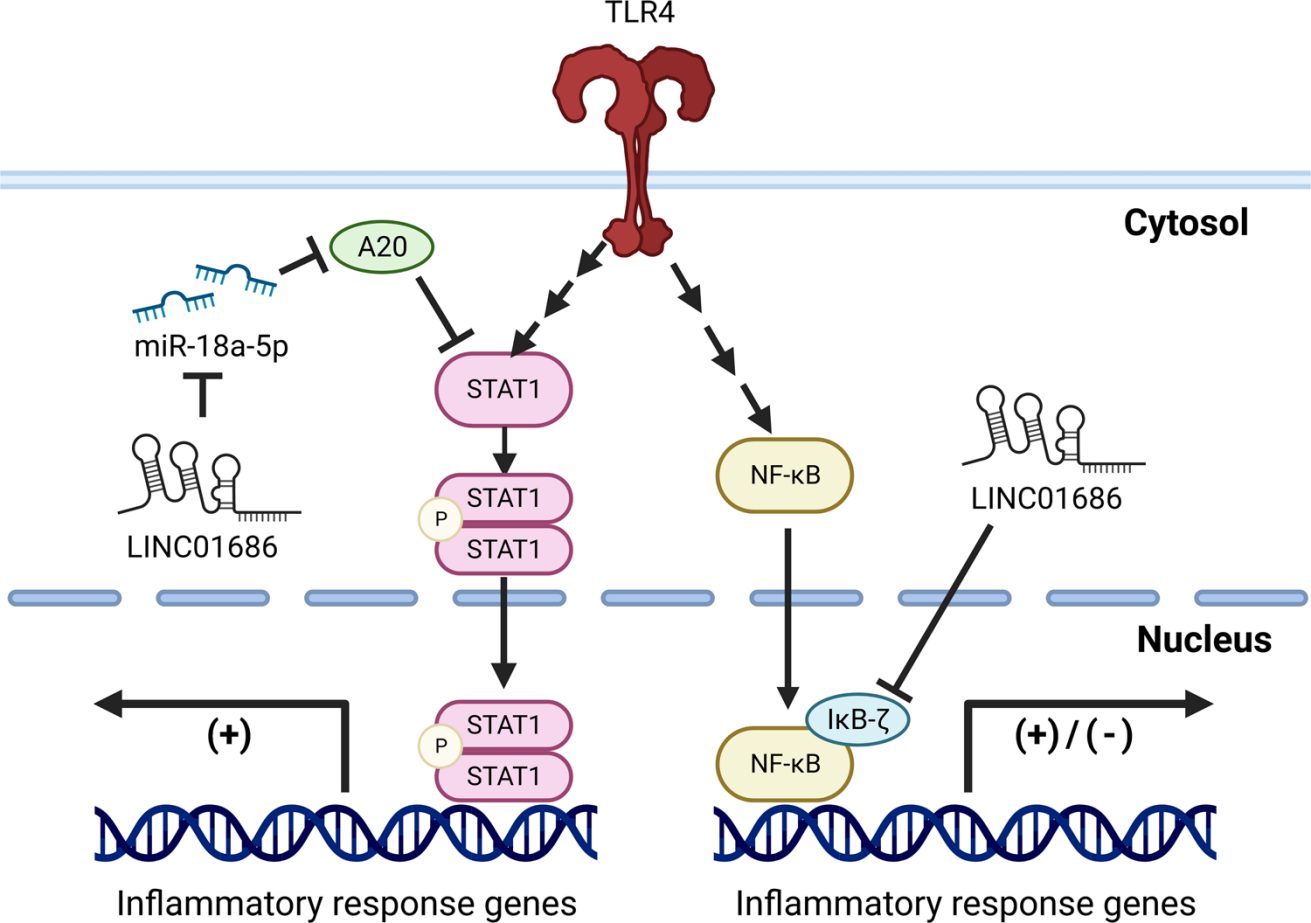
Schematic diagram of the predicted mechanism that LINC01686 regulates STAT‐1 and IκB‐ζ activity. STAT‐1, signal transducer and activator of transcription 1.

LINC01686 exhibits its immunoregulatory function through three main pathways. The first pathway is through the miR‐18a‐5p/A20/STAT1 axis (Figure 7). A20 is a potent anti‐inflammatory enzyme, with deubiquitinase and E3 ligase activities.^30^ Originally, A20 was reported to inhibit the NF‐κB pathway, but recent findings indicate that A20 also inhibits STAT1 activity.^9^, ^10^, ^31^ To inhibit the NF‐κB pathway, A20 removes polyubiquitin chains stabilizing key NF‐κB signaling components and promotes K48‐linked ubiquitination, resulting in proteasomal degradation. A20 further inhibits NF‐κB activity by degrading RIP1 and interacting with NEMO.^7^ In this study, however, LINC01686 attenuation inhibited A20 expression with no significant difference in the expression and phosphorylation of p65. Instead, STAT1 expression and LPS‐induced STAT1 phosphorylation were enhanced by LINC01686 attenuation in an A20‐dependent manner. The current data cannot decipher the cause of this difference in the A20‐mediated regulation of NF‐κB and STAT1 pathways. One possible hypothesis is that other miRNAs affected by LINC01686 offset A20‐mediated NF‐κB regulation. According to databases such as DIANA and miRDB, miR‐302‐3p, one of the potential targets of LINC01686, downregulates the expression of NF‐κB by inhibiting IRAK4 and ZFP91.^32^ The increased NF‐κB expression due to reduced A20 may have been mitigated by increased miR‐302‐3p in LINC01686‐attenuated THP‐1 cells.

The second pathway through which LINC01686 exhibits its immunomodulatory function is through IκB‐ζ, which is a nuclear IκB family protein upregulated by LPS/IL‐1β stimulation (Figure 7). Its expression, like classical IκB proteins, requires NF‐κB‐mediated transcriptional activation, but IκB‐ζ is not degraded upon cell stimulation. IκB‐ζ regulates the DNA binding of NF‐κB without inhibiting nuclear translocation and can selectively activate or interfere with specific NF‐κB activity to regulate genes, including IL‐6, IL‐12 p40, and CSF2.^33^, ^34^, ^35^, ^36^, ^37^ IκB‐ζ also exerts its effect on nucleosome remodeling through the selective regulation of H3K4 trimethylation.^38^ IκB‐ζ serves as a regulator of T cell development^39^ and natural killer cell activation.^40^ It also controls IL‐10 production in B cells^41^ and apoptotic cell death in epithelial cells.^42^ Although LINC01686 attenuation enhanced LPS‐induced increase in IκB‐ζ expression and activity, the mechanism behind this regulation has not been elucidated through current investigation and needs further analyses.

The final immunoregulatory function of LINC01686 is exhibited through mitochondrial dysfunction and subsequent ROS generation. LINC01686 attenuation disrupted mitochondrial membrane potential and increased mitochondrial ROS generation. The fact that antioxidants partially mitigated the LINC01686 attenuation‐induced increase in cytokine secretion demonstrates the involvement of ROS in this process. Therefore, it is speculated that the process by which LINC01686 regulates IL‐6 and IL‐8 secretion involves mitochondrial function. However, the current data cannot explain the mechanism by which LINC01686 affects mitochondrial function and its stability.

Interestingly, LINC01686 attenuation enhanced the LPS‐induced expression of IL‐6 and IL‐8, whereas TNF‐ɑ expression remains unaffected. As the NF‐κB activation process was unaffected by LINC01686 attenuation, TNF‐ɑ expression was also unaffected as it heavily depends on NF‐κB activation. IL‐6 is a cytokine with pleiotropic effects on inflammation, immune response, and hematopoiesis. Activated IL‐6 induces acute phase inflammatory reactions, such as fever and anemia, and promotes B cell differentiation to form antibodies.^43^, ^44^ Dysregulated IL‐6 production causes persistent inflammation and may contribute to the development of autoimmune diseases, such as rheumatoid arthritis.^45^ Although IL‐8 is primarily known as a neutrophil‐specific chemokine, it attracts and modulates the function of several inflammatory cells, including monocytes and T lymphocytes.^46^ IL‐8 has been reported to affect the recruitment and activation of macrophages under various conditions, such as chronic liver diseases and nonalcoholic fatty liver disease.^47^ LINC01686 can be utilized as a therapeutic immunosuppressant in diseases where IL‐6 and IL‐8 activity is crucial.

Current research data have elucidated the regulatory roles of LINC01686 in monocyte/macrophage lineage cells; however, limitations exist. Firstly, the conclusions regarding its function are primarily based on loss‐of‐function mutations, with a notable absence of gain‐of‐function mutations, such as overexpression studies. Additionally, for these results to be deemed significant, it is imperative to establish a correlation between alterations in LINC01686 expression levels and disease pathogenesis. This could involve the analysis of its expression in human disease samples, patient serum, or animal models of disease. Further investigations into these aspects are necessary.

In summary, attenuating LINC01686 expression enhanced ROS production by increasing the total amount of mitochondria and destabilizing the mitochondrial membrane potential. LINC01686 attenuation also promoted the expression and activation of IκB‐ζ and STAT1. STAT1 expression is regulated by LINC01686 via miR‐18a‐5p/A20 axis. The combined action of ROS, IκB‐ζ, and STAT1 is believed to be responsible for enhancing the LPS‐induced expression of IL‐6 and IL‐8.

## AUTHOR CONTRIBUTIONS

Won‐Ha Lee managed the project, provided financial support, and edited the manuscript. Jongwon Baek and Hyeung‐Seob Shin conducted all the experiments and wrote original draft. Kyoungho Suk helped perform the analysis with constructive discussions.

## CONFLICT OF INTEREST STATEMENT

The authors declare no conflicts of interest.

## Data Availability

The data that support the findings of this study are available from the corresponding author upon reasonable request.
